# Determinants of community-based home care service demand among urban older adults in Shanxi, China: a cross-sectional psychological perspective

**DOI:** 10.3389/fpubh.2025.1645632

**Published:** 2025-08-26

**Authors:** Haoran Li, Sijie Sun, Elena Bartomeu-Magaña, Xin Wei

**Affiliations:** ^1^Department of Philosophy, Autonomous University of Barcelona, Barcelona, Spain; ^2^College of Architecture and Art, Taiyuan University of Technology, Taiyuan, China; ^3^Faculty of Information and Communication Sciences, Universitat Oberta de Catalunya, Barcelona, Spain; ^4^Faculty of Geography and History, University of Barcelona, Barcelona, Spain

**Keywords:** community older adult residents, community-based home care, older adult care service demand, Shanxi, a cross-section survey

## Abstract

**Introduction:**

Given China’s large population and the severity of its aging issue, community-based home care has become the primary approach to addressing older adults’ care needs.

**Methods:**

This study combined follow-up visits and questionnaire surveys, using univariate analysis of variance (ANOVA) and multivariate logistic regression to examine the demand for community-based home care services among urban community older adults in Shanxi Province, China.

**Results:**

Logistic regression results identified several significant influencing factors: monthly income, occupation, living conditions, community neighborhood relationships, and community mutual assistance.

**Discussion:**

This study clarifies the characteristics of demand for community aged care services among urban older adults in Shanxi, providing important reference for optimizing such services.

## 1 Introduction

The acceleration of global aging has led to a gradual increase in the proportion of the older adults in the total population of various countries, which not only affects family structures but also poses new challenges to social economic, cultural, and healthcare systems. According to the United Nations, by 2050, the number of people aged 65 and above will reach 2 billion, accounting for over 16% of the global total population (1). In China, data from the 2020 population census shows that there are approximately 264 million people aged 60 and above, making up 18.7% of the total population, and it is projected that the number of people aged 65 and above will reach 200 million by 2050 (2–4). In response to this trend, China has actively promoted community-based aging in place, an innovative model that provides community-based life care and service support for the older adults, enabling them to live a dignified, independent, and secure life in a familiar family environment (5). This model, which emphasizes the autonomous choices and personalized needs of the older adults, is in line with the preference of 98.4% of China’s older adults population for home care (6). It not only aligns with traditional Chinese filial piety values and the emotional attachment of the older adults to their residential and community environments (7–9) but also helps alleviate the pressure of institutional care and the scarcity of nursing resources, given the immature development of China’s nursing home market (10). Additionally, drawing on the successful experience of community-based aging in place from countries like Nordic nations and Japan (11, 12), and leveraging the opportunities brought by intelligent technologies such as telemedicine and smart homes (13–16), China is striving to advance the development of community-based home care.

However, there are deficiencies in the governance of aging in China communities. Despite the promotion of community-based aging in place and the diversification of service forms, the coverage and satisfaction of existing community-based home care services still need to be improved. There may be issues such as inadequate integration of resources, lack of personalized services, and insufficient responsiveness to the diverse needs of the older adults in daily living, medical and health care, and emotional support.

Against this backdrop, this study intends to adopt a combination of quantitative and qualitative methods to conduct a cross-sectional survey on the home care service needs of the older adults in urban communities in Shanxi Province. The main purpose is to comprehensively analyze their needs at multiple levels, collect specific information on their needs for daily living, medical and health care, and emotional support, and assess the coverage and satisfaction of existing services, so as to provide a reference for improving the community-based aging governance in China.

## 2 Subjects and methods

### 2.1 Research subjects

This study selected Shanxi Province in North China as the sample for conducting a cross-sectional study. According to the 2021 7th National Population Census Bulletin, the proportion of the population aged 60 and above in China accounted for 18.70%, in Shanxi Province, the proportion of the population aged 60 and above is 18.92%, which has exceeded the national average (17, 18). To gain a deeper understanding of the existing issues in community-based home care and the challenges and needs faced by older adults in urban communities during the aging process at home. A total of 1,046 participants from Shanxi Province completed the questionnaire survey in this study. The selection criteria for participants included the following inclusion and exclusion criteria. Inclusion criteria: (1) individuals aged 60 and above; (2) older adults residing in urban communities; (3) older adults capable of independently and clearly expressing their personal preferences; (4) older adults willing to cooperate and participate in the research. Exclusion criteria: (1) older adults with mental disorders or their caregivers (Excluding the older adults and caregivers with mental disorders, due to their highly special care needs, this study focuses on the general needs of the general older adults in the community, and the follow-up research will be specific to this group); (2) older adults unwilling or unable to cooperate with the research; (3) older adults whose family members do not consent to their participation in this study. Written informed consent was obtained from all participants.

### 2.2 Methods

A cross-sectional survey was conducted from November 22, 2022, to January 2, 2023. This study employed a combination of online and offline methods based on the practical circumstances during the survey process. To avoid age discrimination resulting from the digital divide, multiple methods were employed for questionnaire collection. Online questionnaires accounted for a significant proportion of the collected responses. These questionnaires were generated using the “QuestionStar” software and were disseminated and shared through older social groups, WeChat moments, social media, and other channels.

### 2.3 Questionnaire design

The first section primarily investigates the respondents’ basic information and demographic characteristics, including residential address, age, gender, marital status, children’s status, educational level, and past occupations. The purpose is to gather insights into the basic profile of the surveyed older individuals.

The second section focuses on investigating the living conditions of the respondents, including their housing situation, daily activities and dietary habits, older adult care preferences, economic situation, and relationships with family members. The objective is to gain an understanding of the challenges and needs faced by older individuals in their daily lives.

The third section primarily investigates the health status of the respondents, including their health conditions such as sleep patterns, chronic illnesses, and mental well-being. It also covers the medical situation of the older, including health management, healthcare expenses, and medical insurance. The objective is to understand the main challenges and needs of the older population regarding their health.

The fourth section primarily evaluates the status of community-based older adult care services, including the level of coverage, user experience, satisfaction level, and the alignment between service offerings and the needs of the older. The objective is to understand the current situation of community-based older adult care services, identify areas for improvement, and explore directions for enhancement.

The fifth section primarily investigates the relationship between the older and the community, the willingness of the older to participate in community-based mutual support for older adult care, and their willingness to use age-friendly applications related to older adult care. The purpose is to understand the feasibility of implementing community-based mutual support for older adult care.

### 2.4 Data analysis

All survey data were analyzed using SPSS 23.0 for statistical analysis. Descriptive statistics were used to report measures such as mean ± SD or percentages (%). Group comparisons were conducted using t-tests and one-way analysis of variance (ANOVA). Multivariate logistic regression analysis was employed to identify influential factors. A significance level of *p* < 0.05 indicated statistically significant differences.

## 3 Results

### 3.1 Population characteristics of urban community older residents

The population characteristics of older residents in urban communities are shown in Table 1. Among the 1,046 participants, more than half of the respondents were female (67.7%) and married (85.4%). The majority of participants had children (98.8%), received retirement pensions (65.74%), and had a level of education no lower than junior high school (84.035%). Most of the respondents had a monthly income greater than 2000 yuan (69.99%), and 78.8% were economically independent. In this survey, the age group of 60–65 accounted for 48.279%, 66–70 accounted for 31.5487%, 71–75 accounted for 11.8546%, 76–80 accounted for 3.7284%, and those over 80 accounted for 4.5889%. Table 1 shows that there are differences in the demand for community-based older adult care among different genders (*p* < 0.01), monthly income levels (*p* < 0.001), education levels (*p* < 0.001), and past occupations (*p* < 0.001). Retired cadres have a higher demand for community-based older adult care, followed by workers and farmers (Table 1).

**Table 1:** Population characteristics of urban community older residents.

Variable	Total (n)	%	Have community-based home care services needs	%
Gender			n	%
Male	338	32.3	250	74.0
Female	708	67.7	581	82.1
χ^2^				9.187
*p*-value				0.002
Age				
60–65	505	48.3	403	79.8
66–70	330	31.5	260	78.8
71–75	124	11.9	95	76.6
76–80	39	3.7	31	79.5
>80	48	4.6	42	87.5
χ^2^				2.643
*p*-value				0.619
Monthly income				
<2000	314	30.0	176	56.1
2001–3,000	211	20.2	109	51.7
3,001–4,000	243	23.2	184	75.7
4,001–5,000	186	17.8	102	54.8
>5,000	92	8.8	60	65.2
χ^2^				36.34
*p*-value				<0.001
Education level				
Elementary school	167	16.0	111	66.5
Junior High School	370	35.4	224	60.5
High School	285	27.2	211	74.0
College	170	16.3	105	61.8
Undergraduate	48	4.6	20	41.7
Postgraduate	6	0.6	2	33.3
χ^2^				28.049
*p*-value				<0.001
Occupation				
Cadres	221	21.1	171	77.4
Employees	467	44.6	344	73.7
Individuals	110	10.5	49	44.5
farming	138	13.2	101	73.2
Unemployed	84	8.0	54	64.3
Other	26	2.5	12	46.2
χ^2^				51.541
*p*-value				<0.001

### 3.2 Assistance situation for urban community-dwelling older in home-based care

Currently, 14.8% of older residents aged 60 and above in urban communities live alone in their daily lives, while 45.3% of older residents prefer to be accompanied by their partners and family members for older adult care. The study found that having children (*p* < 0.001) and living with a spouse (*p* < 0.001) were associated with lower willingness for community-based older adult care. In terms of daily life arrangements, those who receive care from the government or community neighbors have a higher demand for community-based older adult care (*p* < 0.001). Older residents living in welfare institutions have a higher demand for community-based older adult care (*p* < 0.001). Our study also revealed that older adults who expressed a desire to select older adult groups, community neighbors, and volunteers as companions in their old age had a significantly higher demand for community-based home care (*p* < 0.001; Table 2).

**Table 2:** Assistance situation for urban community-dwelling older in home-based care.

Variable	Total (n)	%	Have community home care needs	%
Children				
None	13	1.2	12	92.3
1	327	31.3	228	69.7
2	491	46.9	325	66.2
3	153	14.6	128	83.7
4	51	4.9	42	82.4
≥5	11	1.1	10	90.1
χ^2^				25.952
*p*-value				<0.001
Marital status				
Unmarried	10	1	6	60
Married with spouse	893	85.4	628	70.3
Divorced	32	3.1	22	68.8
Widowed	107	10.2	84	78.5
Other	4	0.4	3	75.0
χ^2^				3.824
*p*-value				0.43
Living arrangement				
Self	109	10.4	87	79.8
Spouse	414	39.6	204	49.3
Children	329	31.5	217	66.0
Relatives	58	5.5	49	84.5
Healthcare professionals, caregivers, volunteers	40	3.8	34	85.0
Government, community, and group care	36	3.4	31	86.1
Older groups, community neighborhoods, volunteers	43	4.1	37	86.0
Other	17	1.6	12	70.6
χ^2^				86.737
*p*-value				<0.001
Willing older companion				
Companions, family members	474	45.3	232	48.9
Healthcare professionals, caregivers, volunteers	420	40.2	158	37.6
Older groups, community neighbors, volunteers	152	14.5	150	98.7
χ^2^				169.142
*p*-value				<0.001
Parent–child relationship				
Very good	730	69.8	477	65.3
Fairly good	203	19.4	172	84.7
Poor	9	0.9	4	44.4
Fair	86	8.2	54	62.8
Very poor	18	1.7	12	66.7
χ^2^				31.997
*p*-value				<0.001
Residence status				
Living alone	155	14.8	123	79.4
Living with partner	696	66.5	342	49.1
Living with children	132	12.6	81	61.4
Welfare sector	35	3.3	33	94.3
Living with a nanny (caregiver)	28	2.7	20	71.4
χ^2^				72.494
*p*-value				<0.001

### 3.3 Home-based care service situation for urban community-dwelling older

We surveyed the status of community-based older adult care services for older residents in urban communities. The results showed that currently, the biggest challenge faced by older people aged 60 and above in terms of older adult care is inadequate funding (70.94%). As for the current national medical and older adult care policies, 53.1% of the participants have some knowledge of them. We found that older people without medical insurance coverage tend to have a lower demand for community-based older adult care, and those who currently do not receive home-based older adult care services from the community also have a relatively lower demand for community-based older adult care (Table 3).

**Table 3:** Home-based care service situation for urban community-dwelling older.

Variable	Total (n)	%	Have community home care needs	%
Medical insurance situation			n	%
Publicly funded medical care	222	21.2	171	77.0
Urban workers’ medical insurance	420	40.2	290	69.0
Urban and rural residents medical insurance	289	27.6	191	66.1
Commercial medical insurance	55	5.3	45	81.8
None	60	5.7	36	60
χ^2^				14.038
*p*-value				0.007
Knowledge of national pension policy				
Yes	555	53.1	454	81.8
No	491	46.9	365	74.3
χ^2^				8.541
*p*-value				0.003
Whether the community provides home care services				
Yes	409	39.1	340	83.1
No	637	60.9	379	59.5
χ^2^				64.732
*p*-value				<0.001

### 3.4 Community mutual aid in the urban area

In urban communities, 42.7% of older residents have lived in the community for over 15 years, 53.3% have harmonious relationships with their neighbors, and 90.2% would turn to their neighbors for help when facing difficulties. Our research found a positive correlation between the demand for older adult care and community factors such as length of time living in the community, neighborly relationships, participation in community activities, support for community mutual aid projects, and participation in community service activities (Table 4).

**Table 4:** Community mutual aid in the urban area.

Variable	Total (n)	%	Have community home care needs	%
Community lifetime				
Less than 1 month	38	3.6	16	42.1
Less than 1 year	41	3.9	17	41.5
1–5 years	232	22.2	143	61.6
6–10 years	159	15.2	116	73.0
10–15 years	129	12.3	96	74.4
More than 15 years	447	42.7	331	74.0
χ^2^				41.3
*p*-value				<0.001
Neighborhood relationship				
Very good	558	53.3	386	69.2
Better	335	32.0	260	77.6
Fair	146	14.0	73	50.0
Not good	7	0.7	1	14.3
χ^2^				55.9
*p*-value				<0.001
Willing to ask for help from neighbors when in trouble				
Yes	944	90.2	684	72.5
No	102	9.8	35	34.3
χ2				62.3
P-value				<0.001
Willing to help the community in any way they can				
Yes	997	95.3	706	70.8
No	49	4.7	13	26.5
χ2				42.6
P-value				<0.001
Whether they want to exist online or offline to solve the problems in the process of aging				
Yes	967	92.4	699	72.3
No	79	7.6	20	25.3
χ^2^				75.0
*p*-value				<0.001
Support for community-based mutual help programs for neighbors				
Support	867	82.9	632	72.9
Fair	172	16.4	92	53.5
Do not support	7	0.7	1	14.3
χ^2^				35.444
*p*-value				<0.001
Participation in service activities provided by community neighborhood support programs				
Willingness	856	81.8	638	74.5
Generally	173	16.6	81	46.8
Reluctant	17	1.6	5	29.4
χ^2^				64.719
*p*-value				<0.001
Willing to age with community neighbors through mutual help				
Willing	809	77.3	619	76.5
Generally	205	19.6	104	50.7
Not willing	32	3.1	6	18.8
χ^2^				92.035
*p*-value				<0.001

### 3.5 Correlation analysis between home-based care service situation and community mutual aid for urban community-dwelling older

The correlation analysis between the status of community-based older adult care services and community mutual aid among urban older residents is shown in the table. We use the Odds Ratio (OR) to measure the strength of association between groups, and the 95% Confidence Interval (95% CI) is used to assess the reliability of the OR. Logistic regression analysis showed that monthly income, previous occupations, living conditions, community neighbors, and community mutual aid all affect the demand for community-based older adult care among older residents (*p* < 0.05; Table 5).

**Table 5:** Correlation analysis between home-based care service situation and community mutual aid for urban community-dwelling older.

Variable	OR	95% Confidence interval	*p*
Monthly income (Ref. below 2000)			
2001–3,000	1.193	0.841–1.649	0.322
3,001–4,000	0.518	0.355–0.756	0.001
4,001–5,000	1.330	0.914–1.934	0.136
≥5,000	0.861	0.527–1.407	0.621
Previous Occupation (Ref. Cadre)			
Employee	1.223	0.839–1.782	0.303
Individual	4.258	2.607–6.954	<0.001
farming	1.253	0.767–2.047	0.378
unemployed	1.900	1.100–3.281	0.028
Other	3.990	1.735–9.177	0.001
Children (Ref. no children)			
1	5.211	0.668–40.620	0.119
2	6.129	0.790–47.542	0.070
≥3	2.052	0.382–11.025	0.401
Residence status (Ref. living alone)			
Living with partner	3.979	2.624–6.033	<0.001
Living with a son or daughter	2.420	1.434–4.085	0.001
Welfare sector	0.233	0.053–1.023	0.048
Living with a nanny (caregiver)	1.538	0.620–3.810	0.333
Does the community provide aging-in-place services (Ref. yes)			
No	3.354	2.477–4.543	<0.001
Will neighbors seek help when in trouble (Ref: Yes)			
No	5.036	3.266–7.765	<0.001
Willing to help the community in any way they can (Ref. Yes)			
No	6.718	3.512–12.853	<0.001
Whether they want to exist online and offline to solve problems in the process of aging (Ref. Yes)			
No	7.694	4.546–13.024	<0.001
The degree of support for the community to carry out neighborhood mutual help programs (Ref. Support)			
General	2.339	1.673–3.269	<0.001
Do not support	16.136	1.932–134.741	0.002
The degree of participation in service activities provided by community neighborhood mutual aid projects (Ref. willing)			
Generally	3.164	2.301–4.350	<0.001
Unwilling	14.118	5.726–34.809	<0.001

## 4 Discussion

Given the enormous population size and severe aging issue in China, Community-based Home Care for the Older may become the primary approach to address the aging population’s needs (19). Moreover, according to the United Nations projections, the population of older individuals aged 65 to 79 in China is expected to increase in the future. Consequently, there will be a growing and increasingly diverse demand for older adult care services (20).

This study investigated the older adult care needs of urban community-dwelling older adults. By gaining an understanding of the home care assistance situation among older residents in urban communities, it can be observed through an analysis of their children’s circumstances that older residents without children have a higher demand for community-based home care, while the majority of those with children have relatively lower demand. Furthermore, among urban community-dwelling individuals aged 60 and above, 14.8% choose to live alone, while 45.3% prefer to live with their partners or family members for their older adult care. This indicates a close relationship between the older’s living choices and their social support networks, as they prefer to have companionship during their later years (21). Among them, older residents living with spouses and family members exhibit lower demand for community-based home care. This may be attributed to the presence of family members who provide companionship and support, reducing the need for additional community-based home care services. On the other hand, older individuals living alone have a relatively higher demand for community-based home care services. Additionally, there is currently a higher demand for community-based home care services from older people who are cared for by the government or their community neighborhoods. This could be due to their lack of family support or the inability of family members to fully cater to their older adult care needs. Furthermore, older residents living in welfare facilities also display higher demand for community-based home care, potentially because of their more vulnerable living conditions, necessitating greater social support. Moreover, the study found that older adults tend to prefer older peers, community neighbors, and volunteers as intended companions for their older adult care, indicating their willingness to receive support and care from the community rather than relying solely on family members. This demand underscores the importance of community-based home care services, as they can provide older individuals with more social opportunities and community support, enhancing their sense of well-being and quality of life (22). Regarding living conditions, the research revealed that 14.8% of urban community-dwelling older residents live alone. Furthermore, older residents living in welfare facilities exhibit higher demand for community-based home care (*p* < 0.001). Additionally, the study also found that older residents receiving care from the government or community-based neighborhood assistance display higher demand for community-based home care (*p* < 0.001). This emphasizes the crucial role of government and community care in meeting the home care needs of older adults. These research findings indicate the significance of family support and government or community-based neighborhood assistance for the home care demand.

Community mutual support can stimulate social engagement and a sense of responsibility among older adults, enriching their lives and enhancing interpersonal relationships, quality of life, and well-being (23, 24, 25). Data analysis revealed a positive correlation between community support and older adult care needs, including factors such as the amount of time spent in the community, neighborly relationships, participation in community activities, support for community mutual aid projects, and engagement in community service activities. A significant proportion of the older residents expressed their willingness to contribute to the community according to their abilities. Additionally, the majority of older adults reported harmonious relationships with their neighbors and a willingness to seek help from them when facing difficulties. These findings highlight the positive impact of community mutual support on the older residents’ care needs. Through community mutual support, older adults can establish positive neighborly relationships and access more community support and services.

By exploring the influence of community mutual assistance on the demand for community-based home care for the older and analyzing a series of variables, it was found that there is a positive correlation between factors such as the duration of the community residence, neighbor relationships, participation in community activities, support for community mutual assistance projects, and participation in community service activities. The research results indicate that over 42.7% of the older residents have been living in the community for more than 15 years, and among this group, 74.0% express demand for community-based home care services. This finding suggests that as older residents spend more time in the community, they are more inclined to choose the home care services provided by the community. The positive correlation between community mutual aid and nursing needs may be due to the increased accessibility of services, and mutual aid networks promote the dissemination of service information and the docking of resources. At the same time, social cohesion is enhanced, and good neighborhood relations enhance the trust and willingness of the older adults to use community services (26).

Furthermore, logistic regression analysis was conducted to explore the association between the provision of community-based home care services and community mutual assistance among older residents in urban areas. The analysis identified several factors that influence the care demands. Firstly, monthly income was found to be moderately associated with the care demands for community-based home care services among older residents. This may be attributed to the fact that older residents with lower incomes require more economic support and services provided by the community, whereas those with higher incomes have more options and resources to address their care needs independently. Secondly, the previous occupation of the older residents also influences their care demands for community-based home care and their level of participation in community mutual assistance. Additionally, the living arrangements of older residents were found to be closely associated with their care demands for community-based home care services and their level of participation in community mutual assistance. This may be because older residents living with family members can receive more support and care from their families but also require more community services and assistance to meet their specific care needs. The availability of community-based home care services and the level of support for neighborly mutual assistance projects significantly impact the care demands of older residents and their participation in community mutual assistance. This suggests that the absence of community-based home care services may lead to more difficulties and challenges for older residents during their care process, making them more reliant on community support and mutual assistance. Moreover, communities with higher levels of support for neighborly mutual assistance projects correspondingly exhibit higher levels of participation in community mutual assistance among older residents. This indicates that neighborly mutual assistance projects in the community can stimulate the willingness of older residents to participate and enhance community cohesion and mutual assistance networks (9). Additionally, the study found that the personal characteristics of older residents also influence their care demands for community-based home care services and their participation in community mutual assistance. The attitudes of older residents toward community mutual assistance and support also impact their care demands for community-based home care services and their level of participation. Older residents who are willing to provide help and support to the community are more likely to require community-based home care services and actively participate in community mutual assistance activities (27).

## 5 Limitation

Despite providing valuable insights into the care demands and community mutual assistance among older residents in urban China, this study has several limitations. Firstly, the sample of the study was limited to older residents in a specific city in northern China, which may restrict the generalizability and applicability of the results to other regions and populations. Secondly, the study relied on cross-sectional survey methods, which have methodological and design limitations. Additionally, the extensive questionnaire length may have led to some participants responding inattentively or providing arbitrary answers, potentially introducing data biases. There may still be a selection bias in the difference in the online/offline ratio, and the older adults with low digital literacy may be underrepresented. Finally, although this study provided some understanding of the care demands and community mutual assistance among older residents in urban China and offered valuable insights for future community-based care services, further research is needed to explore the causal relationships between relevant variables and elucidate the specific mechanisms driving the satisfaction of care demands.

## 6 Conclusion

Our results revealed the demand for community-based home care services and some decision factors among the older residents in urban communities in China. Based on these findings, researchers can develop more targeted community-based care policies and services based on these factors to improve the quality and coverage of community-based home care services. In practice, governments and communities should allocate resources for community-based home care services according to the specific needs of different gender and age groups, aiming to meet the demands of the older and enhance their quality of life. Additionally, in the process of promoting the development of community-based home care services, attention should be paid to the influence of gender and age factors, and more targeted policies and measures should be formulated. Additionally, scientific design of community-based older adult care services and exploring the use of mobile applications to establish community mutual assistance platforms offer valuable data references and innovative approaches for future older adult care services. Through continuous improvement and innovation, we can create a healthier, more convenient, and fulfilling community life for older adults, thereby increasing their happiness and quality of life and contributing positively to the sustainable development of society. Compared with Japan’s diverse community-based older adults care services and the Nordic comprehensive community-based medical-social support system, there is still a gap in the service integration and resource investment of China’s community home-based older adults care, and the results of this study provide a basis for learning from international experience to optimize local services.

## Data Availability

The raw data supporting the conclusions of this article will be made available by the authors, without undue reservation.
