# Correction: Determinants of community-based home care service demand among urban older adults in Shanxi, China: a cross-sectional psychological perspective

**DOI:** 10.3389/fpubh.2025.1694051

**Published:** 2025-09-23

**Authors:** Haoran Li, Sijie Sun, Elena Bartomeu-Magaña, Xin Wei

**Affiliations:** ^1^Department of Philosophy, Autonomous University of Barcelona, Barcelona, Spain; ^2^College of Architecture and Art, Taiyuan University of Technology, Taiyuan, China; ^3^Faculty of Information and Communication Sciences, Universitat Oberta de Catalunya, Barcelona, Spain; ^4^Faculty of Geography and History, University of Barcelona, Barcelona, Spain

**Keywords:** community older adult residents, community-based home care, older adult care service demand, Shanxi, a cross-section survey

In the published article, the details for affiliation 2 were incorrect.

The affiliations for Sijie Sun should be listed as “2,1” instead of “1,2”.

Elena Bartomeu-Magaña should have the affiliation “Faculty of Information and Communication Sciences, Universitat Oberta de Catalunya, Barcelona, Spain”.

Xin Wei should have the affiliation “Faculty of Geography and History, University of Barcelona, Barcelona, Spain”.

Haoran Li and Sijie Sun should be listed as equally contributing authors sharing first authorship.

The correct author details are listed below:

Haoran Li^1^^†^, Sijie Sun^2,1^^†^, Elena Bartomeu-Magaña^3^, and Xin Wei^4*^

^1^Department of Philosophy, Autonomous University of Barcelona, Barcelona, Spain

^2^College of Architecture and Art, Taiyuan University of Technology, Taiyuan, China

^3^Faculty of Information and Communication Sciences, Universitat Oberta de Catalunya, Barcelona, Spain

^4^Faculty of Geography and History, University of Barcelona, Barcelona, Spain

^†^These authors have contributed equally to this work and share first authorship

The original version of this article has been updated.

