# Supplementary material for: Determinants of community-based home care service demand among urban older adults in Shanxi, China: a cross-sectional psychological perspective
Source: Front Public Health. 2025 Aug 26;13:1645632. doi: 10.3389/fpubh.2025.1645632 (PMC12417115; doi:10.3389/fpubh.2025.1645632)
Supplement: Supplementary file 1 [file Data_Sheet_1.pdf]

Questionnaire

Questionnaire on the Needs of Elderly Residents in Urban Communities for Community-Based Home Care Services

Dear respondent:
Hello! Aging has become one of the most significant social concerns. In order to better understand the current situation of elderly home care services and improve the quality of life in old age, we are conducting this survey. The questionnaire is to be completed online. Thank you very much for participating and sharing your views and opinions. The survey is anonymous and your responses will be kept strictly confidential and used solely for research purposes. We sincerely appreciate your support and cooperation! Wish you a happy life!

I. Basic Information

Your residential address:
Shanxi Province ___ City, ___ District, ___ Street, ___ Community [Fill-in-the-blank] *

Please select your age group: [Single choice] *
○ Under 60
○ 60–65
○ 66–70
○ 71–75
○ 76–80
○ Over 80

Your gender: [Single choice] *
○ Male
○ Female

Your current marital status: [Single choice] *
○ Never married
○ Married
○ Divorced
○ Widowed
○ Other _________________ *

How many children do you have? [Single choice] *
○ None
○ 1
○ 2
○ 3

○ 4
○ 5 or more

Your education level: [Single choice] *
○ Primary school or below
○ Middle school
○ High school/Technical school
○ College diploma
○ Bachelor's degree
○ Master's degree or above

Previous occupation(s): (Multiple choice) *
□ Government official
□ Employee
□ Self-employed
□ Farmer
□ Unemployed
□ Other __________________ *

Main sources of income: (Multiple choice) *
□ Savings
□ Pension
□ Financial support from children
□ Support from other relatives
□ Own earnings
□ Minimum living allowance
□ Other __________________ *

Your approximate monthly income (including pension, family support, government subsidies): [Single choice] *
○ ¥2,000 or below
○ ¥2,001–3,000
○ ¥3,001–4,000
○ ¥4,001–5,000
○ Over ¥5,000

Your average monthly living expenses: [Single choice] *
○ ¥500 or below
○ ¥501–1,000
○ ¥1,001–2,000
○ ¥2,001–3,000
○ Over ¥3,000

Aspects of old age life you care most about: (Multiple choice) *
□ Living environment
□ Medical facilities

□ Quality of service
□ Other _________________ *

II. Living Situation
Your current living arrangement: (Multiple choice) *
□ Living alone
□ Living with spouse
□ Living with children
□ Living with spouse and children
□ Welfare institution (e.g., nursing home)
□ Living with caregiver
□ Other _________________ *

Who mainly takes care of your daily life?: (Multiple choice) *
□ Fully independent
□ Spouse
□ Children or grandchildren
□ Relatives or friends
□ Professional caregivers or volunteers
□ Government/community/collective
□ Senior groups or neighbors
□ Others _________________ *

Activities you are unable to perform independently: (Multiple choice) *
□ All can be performed independently
□ Basic exercise (e.g., walking, Tai Chi)
□ Grocery shopping
□ Bathing
□ Using the toilet
□ Getting in/out of bed
□ Dressing (buttons, shoelaces)
□ Using electronic devices
□ Making phone calls
□ Eating
□ Controlling urination/defecation
□ Others _________________ *

Your main daily activities: (Multiple choice) *
□ Walking
□ Housework
□ Caring for grandchildren
□ Senior activity room
□ Reading, listening to radio, health lectures

□ Playing cards/games
□ Others _________________ *

How is your daily diet solved?: (Multiple choice) *
□ Cook for yourself
□ Cooked by others
□ Takeout or restaurant
□ Community canteen
□ Others _________________ *

Preferred elderly care method: (Multiple choice) *
□ Traveling/holiday retirement
□ Home-based care (alone or with children)
□ Home-based care with a nanny
□ Combined home and community care
□ Institutional care
□ Others _________________ *

Whose care and companionship do you hope to receive in old age?: (Multiple choice) *
□ Spouse
□ Relatives
□ Medical professionals
□ Nursing home
□ Caregivers
□ Nanny
□ Volunteers
□ Other seniors
□ Neighbors
□ Community staff
□ Others _________________ *

Monthly financial support provided to children/grandchildren: [Single choice] *
○ None
○ ¥500 or below
○ ¥501–1,000
○ ¥1,001–2,000
○ ¥2,001–3,000
○ Over ¥3,000

Frequency of children's visits: (Multiple choice) *
□ Daily
□ Almost daily
□ 1–2 times a week
□ 1–2 times a month
□ 1–2 times a year

□ None
□ Other __________________ *

Your relationship with your family: [Single choice] *
Very poor ○1 ○2 ○3 ○4 ○5 Very good

III. Health Status
How is your sleep quality? [Single choice] *
○ Very good
○ Good
○ Average
○ Poor

Do you suffer from any chronic diseases? (Multiple choice) *
□ None
□ Hypertension
□ Heart disease
□ Diabetes
□ Cerebrovascular disease
□ Spinal disease
□ Arthritis
□ Respiratory diseases
□ Dementia
□ Gastric problems
□ Liver/gallbladder/bladder disease
□ Cancer
□ Others __________________ *

Usual treatment methods when unwell: (Multiple choice) *
□ Self-medication
□ Community clinic
□ Hospital
□ Family doctor
□ Wait and see
□ Drink water and rest
□ Health supplements
□ Other __________________ *

Your health management methods: (Multiple choice) *
□ Exercise
□ Dietary therapy
□ Health supplements
□ Medication

□ Traditional Chinese medicine
□ Other _________________ *

Monthly health-related spending (medication, treatment, etc.): [Single choice] *
○ ¥500 or below
○ ¥501–1,000
○ ¥1,001–2,000
○ ¥2,001–3,000
○ Over ¥3,000

What type of health insurance do you currently have?: (Multiple choice) *
□ Public medical care
□ Urban employee insurance
□ Urban and rural resident insurance
□ Commercial insurance
□ None
□ Other _________________ *

Does your community have a clinic? [Single choice] *
○ Yes
○ No

Walking time from home to the clinic: (Multiple choice) *
□ Under 5 minutes
□ 5–15 minutes
□ 15–30 minutes
□ 30–60 minutes
□ Over 1 hour

Waiting time at the community clinic: (Multiple choice) *
□ Under 15 minutes
□ 15–30 minutes
□ 30–60 minutes
□ Over 1 hour
□ Never visited
□ Other _________________ *

Communication frequency with children: (Multiple choice) *
□ Daily
□ 2–3 times a week
□ 2–3 times a month
□ 2–3 times a year
□ Other _________________ *

Do you feel lonely? (Multiple choice) *
□ Always

□ Often
□ Sometimes
□ Seldom
□ Never
□ Reason _________________ *

Your hobbies: (Multiple choice) *
□ Learning (reading, lectures, etc.)
□ Art (music, painting, etc.)
□ Sports
□ Traveling
□ Socializing
□ Cooking
□ Others _________________ *

IV. Community-Based Home Care Services
Have you ever learned about national healthcare and elderly care policies? [Single choice] *
○ Yes
○ No (If no, please skip to Question 36)

What suggestions or opinions do you have on current national healthcare and elderly care policies? [Open-ended]
Please share your true thoughts.

Community-based home care, advocated by the government, relies on community organizations to integrate resources and provide diversified services for residents, allowing seniors to enjoy various care services while staying at home. After hearing this, do you think you need community-based home care services? [Multiple choice] *
□ Yes
□ No

Do you have any suggestions for community-based home care? [Open-ended]

Which elderly care model do you prefer? (Multiple choice) *
□ Family-based care
□ Community-based home care
□ Institutional care (nursing homes, senior apartments, etc.)
□ Community mutual assistance care
□ A combination of multiple models (ideal scenario)
□ Other _________________ *

Does your current community provide elderly care services? [Single choice] *
○ Yes
○ No (Please skip to Question 45)

How familiar are you with community-based home care services? [Single choice] *
○ Never heard of it
○ Heard of it, but not familiar
○ Very familiar

Have you ever used community-based home care services? [Single choice] *
○ Yes – Excellent experience
○ Yes – Average experience
○ Yes – Poor experience
○ No – Never used

What services can your community's elderly care institutions provide? (Multiple choice) *
□ Daycare services
□ Nighttime care
□ Emergency call services
□ Meal assistance
□ Recreational activities
□ Mental health services
□ Others _________________ *

Walking time from home to the community service center/activity center: (Multiple choice) *
□ Within 15 minutes
□ 15–30 minutes
□ 30 minutes to 1 hour
□ Over 1 hour
□ Never been

How satisfied are you with the elderly care services in your community? [Single choice] *
○ Very satisfied
○ Satisfied
○ Average
○ Dissatisfied (Reason: _________________)
○ Very dissatisfied (Reason: _________________)

What services do you think should be provided by community elderly care institutions? (Multiple choice) *
□ Senior dining hall/home meal delivery
□ Bathing and grooming

□ Toilet assistance
□ Haircuts
□ Daycare
□ Laundry and ironing
□ Daily shopping
□ Housekeeping
□ Appliance repair
□ House cleaning
□ Health education
□ Escort to hospital
□ In-home medical care
□ Regular physical checkups
□ Establish health records
□ Companionship
□ Psychological counseling
□ Senior activity center
□ Elderly university
□ Cultural/recreational activities
□ Smart device usage guidance
□ Other _________________ *

How much are you willing to pay monthly for the above services? [Single choice] *
○ ¥500 or below
○ ¥501–1,000
○ ¥1,001–2,000
○ ¥2,001–3,000
○ Over ¥3,000

What is your biggest concern or difficulty regarding elderly care? (Multiple choice) *
□ Insufficient funds
□ Lack of dignity
□ Loss of self-care ability
□ Different views with children
□ Family has no time or willingness to care
□ No close friends or companions
□ Lack of entertainment
□ Difficulties in registration, seeing a doctor, or hospitalization
□ Inconvenience for chronic illness treatment
□ Unable to find suitable care facilities
□ Unprofessional or unreliable caregivers
□ Other _________________ *

V. Community Mutual Assistance

How many years have you lived in your current community? [Single choice] *
○ Less than 1 month
○ Less than 1 year
○ 1–5 years
○ 6–10 years
○ 10–15 years
○ Over 15 years

Do you have close friends in the community? [Single choice] *
○ Yes – Many
○ Yes – Some
○ Yes – Only a few
○ No

How is your relationship with your neighbors? [Single choice] *
○ Very good
○ Good
○ Average
○ Poor
○ Very poor

Would you ask your neighbors for help when in difficulty? [Single choice] *
○ Yes
○ No

Are you willing to help your neighbors when able? [Single choice] *
○ Yes
○ No

Have you ever helped your neighbors? [Single choice] *
○ Yes
○ No

Would you like to get to know your neighbors better through community activities? [Single choice] *
○ Yes
○ No

Do you support mutual aid programs in your community? [Single choice] *
○ Yes
○ Neutral
○ No

Are you willing to participate in mutual assistance service activities organized by the community? [Single choice] *
○ Yes

○ Neutral
○ No

Would you like to have an online and offline service platform to help solve difficulties during elderly care? [Single choice] *
○ Yes
○ No

Are you willing to participate in mutual assistance elderly care with your neighbors? (Mutual assistance elderly care emphasizes mutual support among residents as a supplement to community elderly care.) [Single choice] *
○ Willing
○ Neutral
○ Unwilling

Have you ever used or downloaded any community service apps? How was the experience? [Single choice] *
○ Yes – Good experience
○ Yes – Poor experience
○ No

If there was an app that allows mutual help among neighbors, would you download and use it? [Single choice] *
○ Yes
○ No

If there was an app or website designed specifically for the needs and habits of the elderly, would you be willing to learn and use it? [Single choice] *
○ Very willing
○ Neutral
○ Unwilling

The above questionnaire was exported from the Wenjuanxing (WJX) app.

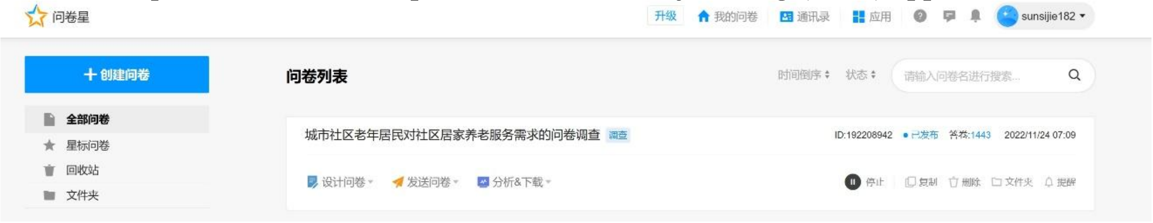

城市社区老年居民对社区居家养老服务需求的问卷调查

尊敬的受访者:
您好！老龄化已成为社会高度关注点之一，为了了解老年人居家养老服务状况，更好的提高老年人晚年生活品质服务，现针对养老需求进行一个调查问卷。本次问卷全部通过网上调查与填写进行。非常感谢您能够作为调查代表参与其中，并提供您的看法与意见。本调查不记名，数据由后台统一处理，我们会对您填写的问卷信息严格保密，问卷结果仅作研究之用。衷心感谢您的支持和协助！祝您生活幸福！

一、基本情况

您居住的地址：

山西省___市、___区、___街道、___社区 [填空题] *

请选择您的年龄段： [单选题] *

○60 岁以下　○60-65 岁　○66-70 岁　○71-75 岁

○76-80 岁　○80 岁以上

您的性别： [单选题] *

○男　○女

您现在婚姻状况 [单选题] *

○从未结过婚

○结婚有配偶

○离异

○丧偶

○其它 ________________ *

您有几个子女？ [单选题] *

○没有子女

○1 个

○2 个

○3 个

○4 个

○5 个及以上

您的受教育程度 [单选题] *

○小学及以下

○初中

○高中/中专

○大专

○本科

○研究生及以上

您曾从事的职业（可多选） [多选题] *

□干部

□职工

□个体经营

□务农

□无业

□其它 ________________*

您现在的经济来源主要是（可多选） [多选题] *

□积蓄

□退休金/养老金

□子女赡养

□其他亲属的补贴

□自己的劳动收入

□享受国家最低生活保障

□其它 ________________*

您的月收入约为（包括退休金、子女支持、政府补贴等全部收入） [单选题] *

○2000 元及以下

○2001-3000 元

○3001-4000 元

○4001-5000 元

○5001 元以上

您每月平均的生活支出约为 [单选题] *

○500 元及以下

○501-1000 元

○1001-2000 元

○2001-3000 元

○3000 元以上

您最关注晚年生活的哪些方面（可多选） [多选题] *

□养老环境

□医疗设施

□服务质量

□其他 ________________*

二 生活情况

您现在居住情况（可多选） [多选题] *

□独居

□与伴侣一起居住

□与儿女一起居住

□与爱人和儿女一起居住

□福利部门（如敬老院等）

□与保姆（其他照料者）同住

□其它 ________________*

您目前的生活主要是由谁来照料？（可多选） [多选题] *

□完全自己生活

□老伴

□子女、孙子孙女

□亲戚或朋友

□专业医护人员、护工、志愿者

□政府、社区、集体照料

□老年群体、社区邻里、志愿者

□其他人 ________________*

您的日常无法独立完成的活动包括（可多选） [多选题] *

□以下均可独立完成

□基本锻炼（散步、拍腿、太极等）

□购物、买菜

□洗澡

□如厕（擦净、整理衣裤、冲水）

□上下床和其他室内简单活动

□穿衣（系鞋带、纽扣）

□使用手机等电子设备

□接打电话

□吃饭

□能够控制大小便

□其他 ________________*

您一天内从事的主要活动为？（可多选） [多选题] *

□散步

□家务劳动

□照顾下一代

□老年活动室

□看书看报、听广播、听健康讲座

□棋牌娱乐

□其他 ________________

您日常饮食怎么解决？（可多选） [多选题] *

□自己做

□他人给做

□外卖或饭店

□社区食堂

□其它 ________________*

您希望通过何种方式养老？（可多选）[多选题] *

□旅游、度假养老

□居家养老（独自养老/与子女生活养老）

□居家养老（雇佣保姆照顾自己生活）

□居家养老＋社区养老

□养老机构养老

□其他 ________________*

您养老过程中希望得到哪一方的照顾与陪伴？（可多选）[多选题] *

□伴侣

□亲人

□专业医护人员

□养老院

□护工

□保姆

□志愿者

□其他老年人

□社区邻里

□社区工作人员

□其他 ________________*

您每月支持子女、孙子女的支出约为 [单选题] *

○没有

○500 元及以下

○501-1000 元

○1001-2000 元

○2001-3000 元

○3000 元以上

您子女探望频率约为 [多选题] *

□每天

□几乎每天

□一周一两次

□一个月一两次

□一年一两次

□无

□其它 _________________*

您与家人的关系如何？ [单选题] *

非常不好 ○1 ○2 ○3 ○4 ○5 非常好

三 健康情况

您的睡眠状况如何 [单选题] *

○很好

○较好

○一般

○较差

您患有哪些慢性病?（可多选） [多选题] *

□无

□高血压

□心脏病/冠心病

□糖尿病

□脑血管病（含中风）

□腰/颈椎病

□关节炎

□支气管炎或其他呼吸道疾病

□老年痴呆症

□胃病

□肝脏、胆囊或膀胱类疾病

□癌症

□其他 ________________

身体不适时一般采取什么样的治疗方法？(可多选） [多选题] *

□到药店买药自治

□社区诊所

□直接去医院就医

□家庭医生

□先吃几天药不见好转再去医院

□多喝水过两天就好

□吃保健品

□其他 ________________*

您平时用哪些健康管理方法？（可多选） [多选题] *

□运动

□饮食疗法

□保健品

□药物

□传统中医疗法

□其他 ________________*

为了维持健康（药品、医疗等），您每月的平均支出约为 [单选题] *

○500 元及以下

○501-1000 元

○1001-2000 元

○2001-3000 元

○3000 元以上

目前您的医疗保险情况属于下列哪种情况?（多选） [多选题] *

□公费医疗

□城镇职工医疗保险

□城乡居民医疗保险

□商业医疗保险

□没有保险

□其它 ________________*

您所居住的社区是否有社区医院？ [单选题] *

○有

○没有

您从家到社区医院所花费的时间?（如果步行） [多选题] *

□5 分钟以内

□5 分钟-15 分钟

□15 分钟-30 分钟

□30 分钟-1 个小时

□1 个小时以上

您在社区医院看病需要排队等候时间为多久? [多选题] *

□15 分钟以内

□15 分钟-30 分钟

□30 分钟-1 个小时

□1 个小时以上

□没有去过

□其它 _________________*

您跟子女的沟通频率约为 [多选题] *

□每天

□每周 2～3 次

□每月 2～3 次

□每年 2～3 次

□其他 _________________*

您是否感到孤独? [多选题] *

□总是

□经常

□有时候

□很少

□从不

□原因 ________________*

您有哪些兴趣爱好（可多选） [多选题] *

□学习（看书、看报、听讲座等）

□艺术（音乐、美术等）

□体育（打球、锻炼等）

□旅游

□交友（社交、聊天、打牌等）

□下厨

□其他 ________________*

四、社区居家养老服务状况

对于国家医疗养老政策有没有了解过？ [单选题] *

○有

○没有 (请跳至第 36 题)

对于目前国家医疗养老政策您有什么建议和看法？ [填空题]
可根据自己的真实想法填写

____

社区居家养老，是一种在政府的倡导下，以社区组织为依托，整合社区内的资源，面向居住区内居民提供的，以满足居民多方面需求的服务，其最大的特点是老人在家也能享受到多方面的养老服务，听完介绍后，您觉得您是否需要社区提供居家养老服务? [多选题] *

□需要

□不需要

对社区居家养老有什么建议吗？ [填空题]

____

下面的养老模式中，您更倾向哪种养老模式?（可多选） [多选题] *

□家庭养老

□社区居家养老

□机构养老（敬老院、养老公寓等）

□社区互助养老

□以上多种模式相结合（理想结合方式）

□其他 _________________*

您目前居住的社区是否提供相关的养老服务？ [单选题] *

○是

○否 (请跳至第 45 题)

您对社区居家养老服务的了解程度 [单选题] *

○没有听过

○听过，不太了解

○很了解

您是否体验过居家养老上门服务？ [单选题] *

○是 体验完美

○是 体验一般

○是 体验很差

○否 没有体验过

您所在社区的养老服务机构能够提供哪些服务？（可多选） [多选题] *

□日间照料服务

□夜间照料服务

□呼叫服务

□助餐服务

□文娱活动

□心里健康服务

□其它 _________________*

从家到社区服务中心（活动中心）所花费的时间?（步行） [多选题] *

□15 分钟以内

□15 分钟-30 分钟

□30 分钟-1 个小时

□1 个小时以上

□没去过

您对您所在社区的养老服务机构是否满意？ [单选题] *

○非常满意

○比较满意

○一般满意

○不满意（可说明原因） _________________

○非常不满意（可说明原因） _________________

您认为社区的养老机构应该提供以下哪些服务（可多选）？ [多选题] *

□老年食堂/送餐

□沐浴/洗护

□协助如厕

□理发

□日间托管照料

□洗衣熨烫

□日常采购

□家政服务

□维修电器

□打扫清洁

□健康知识宣讲

□陪同就医

□上门诊疗/护理

□定期体检

□简历健康档案

□聊天解闷

□心理疏导

□老年活动中心

□老年大学

□文体娱乐活动

□智能设备使用指导

□其他 ________________*

您愿意为以上服务项目每月支付多少钱？ [单选题] *

○500 元及以下

○501-1000 元

○1001-2000 元

○2001-3000 元

○3000 元以上

您遇到的或担心的最大的养老困惑是什么？（可多选） [多选题] *

□养老经费不足

□没有尊严

□生活不能自理

□与子女的观念不同

□家人没时间，或不愿意照顾

□亲友不在身边，无人陪伴

□缺少、没有娱乐活动

□挂号难、看病难、住院难

□慢性病治疗经常往返医院不方便

□找不到合适的养老机构

□护理人员不专业、好的靠谱的护理人员难找

□其它 _________________*

五、社区互助

您在现在的社区住了多少年？ [单选题] *

○1 个月以下

○1 年以下

○1-5 年

○6-10 年

○10-15 年

○15 年以上

您所居住的社区中是否有您关系密切的朋友： [单选题] *

○是 有很多

○是 有一些

○是 只有很少

○没有

您和邻里之间关系如何？ [单选题] *

○非常好

○较好

○一般

○不好

○很差

遇到困难你会想邻里求助吗 [单选题] *

○是

○否

您是否愿意为您社区的邻里提供力所能及的帮助？ [单选题] *

○是

○否

你曾经帮助过您的邻居吗？ [单选题] *

○是

○否

您是否希望通过一些活动，多了解自己的邻居，改善邻里关系？ [单选题] *

○是

○否

您支持社区开展邻里互助项目吗？ [单选题] *

○支持

○一般

○不支持

您愿意参加社区邻里互助项目提供的服务活动吗？ [单选题] *

○愿意

○一般

○不愿意

您是否希望有一种方式，可以通过线上平台和线下服务，帮助您解决在养老过程中遇到的需求和困难？ [单选题] *

○是

○否

您愿意与社区邻里通过互助方式进行养老吗？
（互助养老是一种全新的养老模式，作为社区养老的补充，互助养老更强调普通居民间相互的帮扶与慰藉。） [单选题] *

○愿意

○一般

○不愿意

您是否使用或下载过社区服务类的 APP？使用体验如何？ [单选题] *

○是，体验很好

○是，体验不好

○否

如果有一款 APP，可以使你和邻居互帮互助，你是否愿意下载并使用呢? [单选题] *

○是

○否

如果有一款专门针对老年人的需求和使用习惯而设计的应用软件（APP 或网站），您是否会愿意下载并学习使用它呢？ [单选题] *

○很愿意

○一般

○不愿意

问卷导出自 问卷星
